# Neuroprotective Role of PGC-1α Against Oxidative Stress and Mitochondrial Dysfunction in Chronic Pain Pathophysiology: A Review

**DOI:** 10.3390/biomedicines14102304

**Published:** 2026-10-10

**Authors:** Mubarak Hussaini Ahmad, Khalid Garba Mohammed, Yau Adamu, Mustapha Mohammed, Abubakar Ibrahim Jatau, Ismaeel Yunusa

**Affiliations:** 1 Department of Environmental Health Sciences, School of Public Health, University of Michigan, Ann Arbor, MI 48109, USA; 2 School of Pharmacy, Newcastle University, Newcastle upon Tyne NE1 7RU, UK; khalid.garba@newcastle.ac.uk; 3 School of Life Sciences, Pharmacy and Chemistry, Kingston University London, Penrhyn Road, London KT1 2EE, UK; 4 Department of Pharmaceutics and Industrial Pharmacy, Ahmadu Bello University, Zaria 81001, Nigeria; 5 Department of Environmental and Occupational Health, Kate Marmion School of Public Health, The University of Texas at San Antonio, San Antonio, TX 78229, USA; adamuy@uthscsa.edu; 6 Department of Pharmacology and Therapeutics, Bayero University, Kano 700282, Nigeria; 7 Biomedical Research Center, QU Health, Qatar University, Doha P.O. Box 2713, Qatar; mohammedm@qu.edu.qa; 8 School of Pharmacy and Pharmacology, University of Tasmania, Hobart, TAS 7005, Australia; pharmjt@gmail.com; 9 Department of Clinical Pharmacy and Outcomes Sciences, University of South Carolina College of Pharmacy, Columbia, SC 29208, USA

**Keywords:** chronic pain, PGC-1α, mitochondrial dysfunction, oxidative stress, neuroinflammation, analgesic mechanisms

## Abstract

Chronic pain imposes a significant burden on global healthcare systems, and existing pharmacological interventions often provide inadequate pain management. Long-term opioid therapy for chronic pain has contributed to the ongoing global opioid crisis, and its utilization is restricted by severe adverse effects including tolerance, addiction risks, and fatal respiratory suppression. Available evidence highlights the role of oxidative damage and impaired mitochondrial function in chronic pain across musculoskeletal, neuropathic, cancer, and opioid-induced pain models. However, recent preclinical studies have shown that peroxisome proliferator-activated receptor gamma coactivator-1α (PGC-1α) has the potential to provide effective chronic pain management and to suppress adverse outcomes of chronic opioid therapy. PGC-1α acts by coordinating mitochondrial renewal, redox balance, and neuroinflammation within neural pathways. Its suppression drives mitochondrial dysfunction and pain chronification. Therefore, advancing research into the role of PGC-1α in chronic pain management could uncover effective therapeutic interventions with safer clinical outcomes. However, comprehensive reviews that integrate mechanistic evidence on PGC-1α from multiple chronic pain models—with drug-repurposing implications—remain limited in the literature. This narrative review synthesizes evidence on PGC-1α involvement in chronic pain and examines potential therapeutic interventions through the modulation of PGC-1α as a novel neuroprotective target. Activation of PGC-1α has been shown to attenuate mitochondrial abnormalities and pain hypersensitivity in several preclinical models. Natural compounds, repurposed drugs, and synthetic small-molecule compounds targeting PGC-1α have shown promise as bioactive agents for modulating mitochondrial dysfunction, oxidative stress, and neuroinflammation. However, most available data are preclinical and highlight a translational gap between experimental findings and clinical application across various nociceptive models. Therefore, additional investigation is required to clarify tissue-specific pathways, optimize PGC-1α pharmacological activators, and establish the safety and translational relevance of modulating PGC-1α in chronic pain.

## 1. Introduction

Pain encompasses a distressing neurosensory and psychological state characterized by established or possible tissue impairment [1]. Previous studies have shown that clinical control of severe chronic pain has relied on mu-opioid receptor agonists such as morphine [2]. However, extended opioid therapy is associated with hyperalgesia, addiction liability, and lethal respiratory hypoventilation, which poses risks to many patients [3]. These therapeutic setbacks have encouraged researchers to investigate alternative opioid strategies, including preclinical testing of delta and kappa receptor agonists [4,5]. However, studies have demonstrated some shortcomings related to kappa- and delta-opioid receptors [6,7]. For example, kappa receptor agonists have been limited by dysphoric and psychotomimetic effects, whereas certain delta-opioid receptor agonists are known to provoke convulsant behavior [6,7]. Thus, the development of non-opioid therapeutic alternatives for chronic pain management remains essential [8].

Non-opioid adjuncts, notably gabapentinoids, tricyclic antidepressants, and serotonin-norepinephrine reuptake inhibitors, offer alternative options to overcome the limitations of conventional opioids [8]. However, these options remain suboptimal and are limited by adverse effects [9,10]. Specifically, duloxetine carries a risk of cardiovascular adverse events, whereas gabapentinoids can cause fatigue, ataxia, and have misuse potential [9,10]. Furthermore, minocycline, which has been explored for managing neuroinflammatory responses in persistent lower back pain by inhibiting glial activation, can cause tooth discoloration with prolonged use [11,12].

Chronic pain arises from peripheral and central sensitization, abnormal ion channel and neurotransmitter signaling, glial activation, and neuroimmune remodeling that modifies synaptic function [13,14]. Recent evidence suggests that management of chronic pain could target mitochondrial dysfunction and neuroimmune responses [13,15]. Persistent oxidative damage and disruption of cellular bioenergetics are hallmark features of chronic mitochondrial dysfunction, thereby contributing to neuronal hyperexcitability as well as central pain sensitization [15]. Many currently available analgesics relieve pain by attenuating nociceptive signaling [16]. However, they do not address the underlying mitochondrial and redox abnormalities implicated in chronic pain. At high doses or with extended use, analgesics such as non-steroidal anti-inflammatory drugs (NSAIDs) and opioids tend to impair mitochondrial function and increase oxidative damage [2,17,18]. Given the central role of mitochondrial dysfunction and oxidative stress in chronic pain, there is a growing need to identify upstream regulators of mitochondrial biogenesis as potential therapeutic targets. However, a major research gap remains, as standard analgesics are limited in their ability to restore these upstream pathways [19].

Peroxisome proliferator-activated receptor gamma (PPARγ) coactivator 1-alpha (PGC-1α) regulates mitochondrial generation and antioxidant pathways that modulate redox and inflammatory signaling in persistent pain conditions [20,21]. PGC-1α also controls neuronal excitability and glial activation during nerve-injury-induced pain [22,23,24]. However, despite growing evidence implicating PGC-1α in various pain-related mitochondrial dysfunctions, its therapeutic potential as a non-opioid target has not been explored for translational potential. This review synthesizes information from the recent literature on how PGC-1α modulates mitochondrial dysfunction and cellular oxidative damage in neuropathic, musculoskeletal, cancer-related, and opioid-induced hyperalgesia models [21,25,26]. It highlights the biological functions of PGC-1α and its role in the pathogenesis of persistent pain [24]. The review also explores targeting this coactivator using natural compounds and novel small-molecule activators or stabilizers as therapeutic approaches aimed at restoring mitochondrial integrity and mitigating oxidative burden [27,28]. This evidence positions PGC-1α as a promising target of mitochondrial and redox homeostasis in chronic pain, with considerable potential to drive the development of mechanism-based, non-opioid analgesics. Finally, the review also highlights the translational gaps that need to be addressed for clinical applications.

## 2. How Mitochondrial Dysfunction and Oxidative Burden Drive Chronic Pain

Chronic pain arises from complex cellular and molecular alterations within both central and peripheral neuronal networks [29]. Neuronal mitochondrial dysfunction reduces cellular respiration and increases reactive oxygen species (ROS) production. This eventually contributes to sensory hyperexcitability and neuroinflammation [30]. Oxidative stress intensifies peripheral nociceptor sensitization and glial activation that sustain pain chronification [29]. ROS also disrupt ion channels, disturb inhibitory synaptic transmission, and alter monoaminergic signaling [31,32].

**Figure 1:**
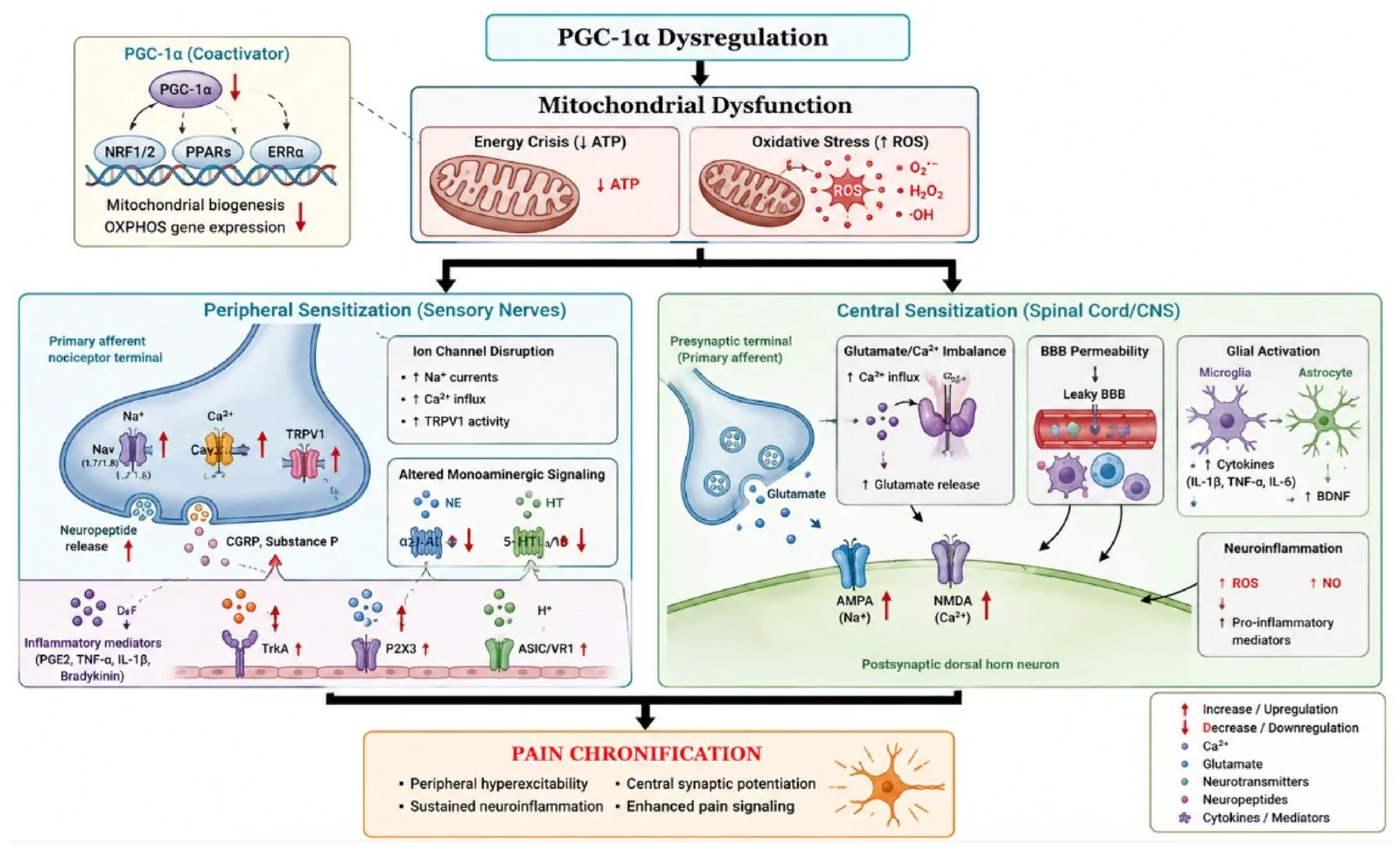
Pathophysiological pathways linking PGC-1α dysregulation and mitochondrial dysfunction to pain chronification. ATP, adenosine triphosphate; BBB, blood–brain barrier; CNS, central nervous system; PGC-1α, peroxisome proliferator-activated receptor-gamma coactivator 1-alpha; ROS, reactive oxygen species.

Within peripheral tissues, mitochondrial bioenergetic failure and oxidative stress activate sensory nerves and increase nociceptive signaling [33]. In the central nervous system (CNS), mitochondrial dysfunction also contributes to central sensitization and neuroinflammation [34,35]. Bioenergetic deficits within the spinal dorsal horn disrupt glutamate and calcium homeostasis, promoting excitotoxic nociceptive transmission [36,37]. Mitochondrial oxidative stress disrupts the blood–brain barrier integrity by altering junctional protein complexes and increasing permeability, which facilitates neuroinflammation and contributes to central sensitization in persistent pain [35,38,39,40]. A reduction in PGC-1α expression correlates with mitochondrial ROS generation within chronic pain in experimental models [19,21,41]. Figure 1 illustrates the cellular mechanisms by which altered PGC-1α signaling contributes to mitochondrial dysfunction and persistent pain.

## 3. Induction of Mitochondrial Toxicity by Analgesics and Adjuvants

Some commonly used analgesics and adjuvants increase mitochondrial injury and ROS production, further reinforcing the need for mitochondria-targeted interventions [42,43,44]. For instance, NSAIDs such as diclofenac and indomethacin disrupt mitochondrial membrane potential and induce bioenergetic stress [45,46,47]. These mechanisms additionally elicit mitochondrial permeability and oxidative stress, which may culminate in apoptotic degradation [48,49]. In addition, indomethacin impairs PGC-1α signaling and triggers mitochondrial dysfunction within gastric cancer cells [50].

Chronic morphine exposure causes mitochondrial dysfunction, ROS accumulation, and morphine tolerance [51,52]. Opioids also increase mitochondrial DNA (mtDNA) release in immune cells to promote nuclear factor kappa B (NF-κB) activation [53,54,55]. Fentanyl and methadone disrupt mitochondrial morphology and reduce neuronal viability in neuronal models [56]. Consistently, PGC-1α prevents opioid-induced mitochondrial impairment [26].

**Figure 2:**
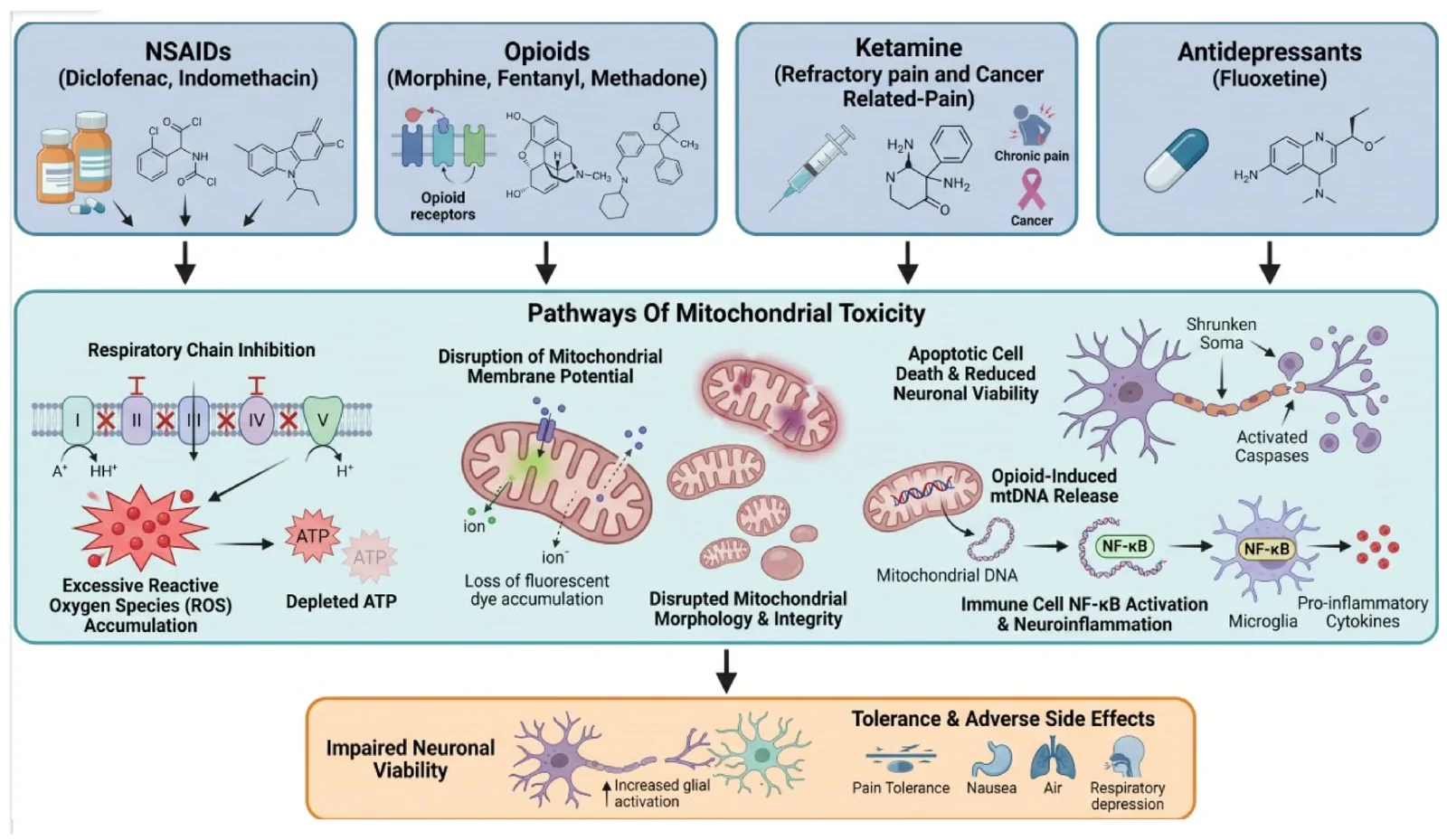
Summary of mitochondrial toxicity and oxidative stress induced by common analgesics and adjuvants. ATP, adenosine triphosphate; mtDNA, mitochondrial DNA; NF-κB, nuclear factor kappa B; NSAIDs, non-steroidal anti-inflammatory drugs; ROS, reactive oxygen species.

As an adjuvant for refractory neuropathic and cancer-related pain, ketamine disrupts mitochondrial homeostasis by depleting ATP and compromising mitochondrial integrity [57,58,59]. These mitochondrial abnormalities could be associated with decreased hippocampal PGC-1α protein expression in neurobehavioral deficits [60]. Similarly, impairment of mitochondrial function may play a role in the side effects associated with adjunctive antidepressants prescribed for chronic pain [61]. Fluoxetine, for instance, downregulates mitochondrial ATP-production pathways in prefrontal cortex interneurons, suggesting an alteration in cellular bioenergetics [62]. These findings indicate that oxidative stress and mitochondrial dysfunction represent an important mechanism underlying drug-induced cellular stress and the adverse effects associated with opioids, indomethacin, ketamine, and antidepressant adjuvants [45,46,47,49,50,51,52,54,56,59,61,62]. The shared involvement of mitochondrial dysregulation highlights the potential role of regulating mitochondrial homeostasis as a promising therapeutic strategy [63]. Therefore, targeting PGC-1α, a key regulator of mitochondrial biogenesis and homeostasis, could help mitigate the cellular toxicity associated with these analgesic agents. Figure 2 summarizes the mitochondrial toxicity associated with commonly used analgesics and adjuvants.

## 4. Regulatory Signaling and Biological Functions of PGC-1α in Chronic Pain

PGC-1α is a central co-activator of transcription that governs mitochondrial formation, oxidative processes, and neuroinflammation [64]. It responds to epigenetic changes by recruiting histone acetyltransferases and the mediator complex to transcriptional complexes [65]. PGC-1α is highly enriched in tissues with elevated energy demands, specifically cardiac muscle, skeletal fibers, and brown adipose tissue [64]. In the CNS, PGC-1α supports neuronal resilience by promoting oxidative phosphorylation and endogenous antioxidant defenses [64]. Studies have linked disrupted PGC-1α activity to neurodegenerative disorders and persistent pain conditions [23,66]. This evidence suggests that PGC-1α may help maintain protective metabolic plasticity in nociceptive circuits [41,67]. Impaired regulation of PGC-1α contributes to both neuropathic pain and neuroinflammation [68,69].

PGC-1α function is controlled via AMP-activated protein kinase (AMPK) alongside SIRT1 as energy-sensing pathways [64,70]. When cells experience sudden energy deficits such as during fasting, physical exertion, or oxygen deprivation, AMPK phosphorylates PGC-1α [64,71]. This phosphorylation event primes PGC-1α for subsequent deacetylation and activation by SIRT1 [64,70,71]. These modifications stimulate PGC-1α, promoting its transcriptional coactivation in the nucleus and establishing the AMPK/SIRT1/PGC-1α pathway as a core regulator of the cellular energy balance [64,70,71].

Following post-translational activation, PGC-1α engages nuclear respiratory factors (NRF-1/NRF-2) to drive the expression of mitochondrial transcription factor A (TFAM) [64,71]. TFAM then enters the mitochondrial matrix, where it regulates mtDNA transcription and maintains oxidative phosphorylation [64,71]. PGC-1α also interacts with PPARs and estrogen-related receptors (ERRs) to regulate the respiratory chain assembly alongside fatty acid oxidation pathways [20]. This cascade helps replace damaged mitochondria in neural tissue, protects cellular metabolism, and maintains the mitochondrial energy demand [20]. These processes suggest that PGC-1α supports synaptic function in response to stress and persistent nociception [67,72].

**Figure 3:**
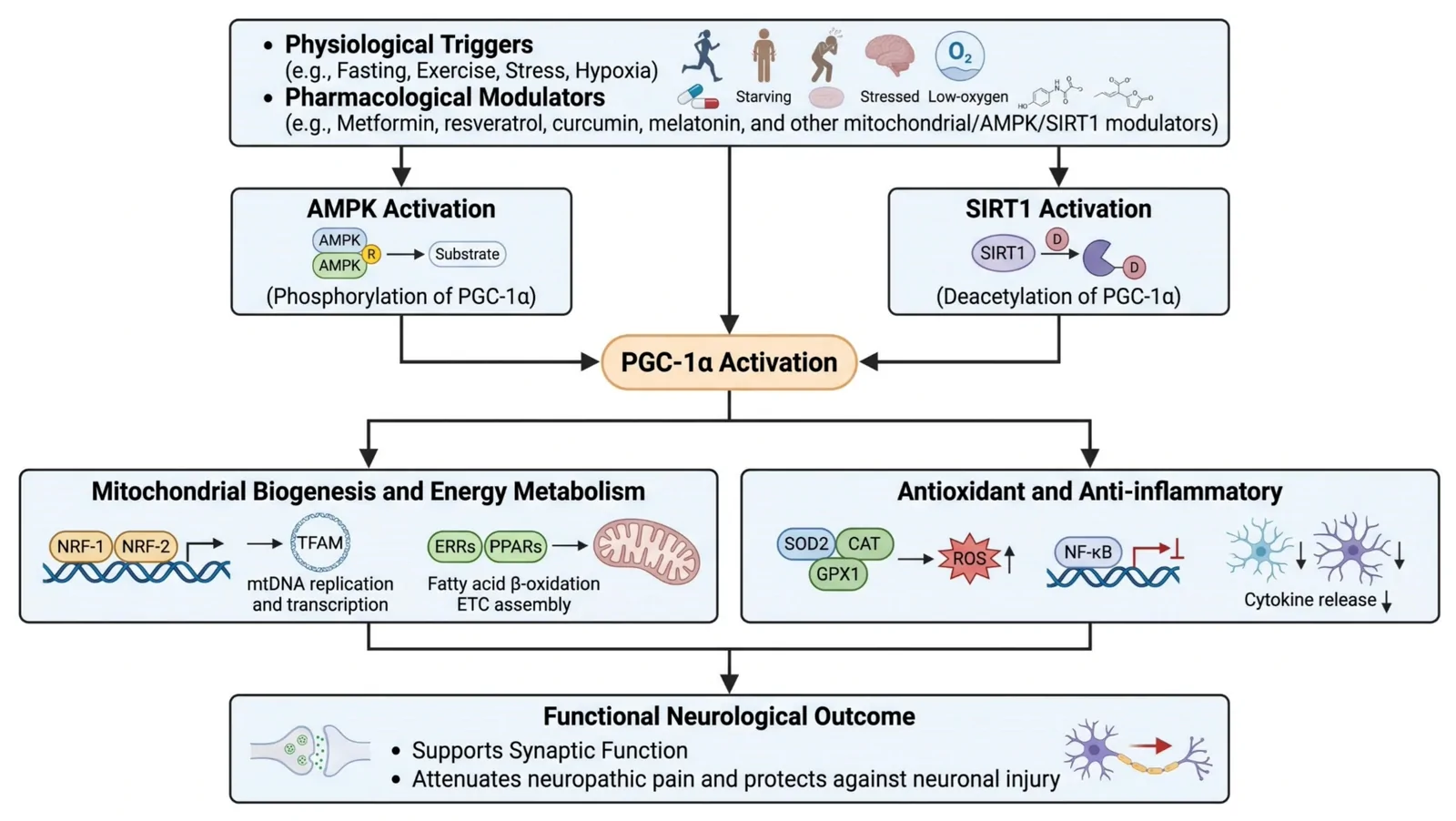
The upstream activation and downstream neuroprotective pathways of PGC-1α in Chronic Pain. AMPK, AMP-activated protein kinase; CAT, catalase; ERRs, estrogen-related receptors; ETC, electron transport chain; GPX1, glutathione peroxidase 1; mtDNA, mitochondrial DNA; NF-κB, nuclear factor kappa B; NRF-1/2, nuclear respiratory factors 1 and 2; PPARs, peroxisome proliferator-activated receptors; ROS, reactive oxygen species; SIRT1, sirtuin 1; SOD2, superoxide dismutase 2; TFAM, mitochondrial transcription factor A.

Furthermore, PGC-1α drives CNS defenses against oxidative stress by upregulating catalase (CAT), superoxide dismutase 2 (SOD2), and glutathione peroxidase 1 (GPX1), thereby restricting ROS accumulation [64,73]. PGC-1α also attenuates neuroinflammatory responses in astrocytes and microglia by suppressing the NF-κB axis and diminishing key cytokines [73]. In CNS models, PGC-1α preserves neuronal survival by limiting oxidative stress and inflammation, while its downregulation promotes neuronal damage [67]. The regulatory signaling pathways and protective biological functions of PGC-1α in chronic pain are summarized in Figure 3.

## 5. Experimental Evidence for PGC-1α in Different Chronic Pain Conditions

Experimental studies using rodent pain models, cultured cells, and human tissue samples indicate that PGC-1α promotes mitochondrial biogenesis alongside cellular redox balance and neuroimmune homeostasis in chronic pain [71,74,75,76]. Conversely, PGC-1α downregulation triggers mitochondrial dysfunction, increased ROS, glial activation, and pain chronification [71,75]. Restoration of PGC-1α signaling reverses mitochondrial quality control deficits while mitigating nociceptive behaviors [75,77,78]. However, existing studies have found that PGC-1α pain-modulating mechanisms vary across chronic pain conditions, depending on tissue, cell type, and pathology. For example, PGC-1α limits cortical hyperexcitability [24], preserves cartilage integrity [79,80], and prevents muscle wasting in osteoarthritis [79]. In peripheral nerve injury, PGC-1α supports mitochondrial resilience in spinal sensory neurons and regulates microglial polarization [21,76]. In chemotherapy and diabetes-induced neuropathic pain, PGC-1α protects neural and peripheral nerves against mitochondrial dysfunction and oxidative injury [80,81,82,83,84]. PGC-1α reduces neuroinflammation and microglial polarization, while preserving GABAergic interneurons in cancer bone pain [25,85]. In fibromyalgia, PGC-1α restores mitochondrial redox balance in muscle and hippocampal tissues [86,87], while in chronic headaches, it protects mitochondrial function and reduces trigeminal neuroinflammation [74,88,89]. Similarly, PGC-1α agonists counteract opioid-induced mitochondrial dysfunction and spinal apoptosis [26,90]. Thus, restoration of PGC-1α signaling could alleviate chronic pain through tissue- and mechanism-specific biological effects. Given that these biological effects vary across different pain conditions, evidence for PGC-1α in chronic pain subtypes is synthesized in the following sections.

### 5.1. Musculoskeletal Pain

Aging-associated pain chronification reflects both peripheral tissue degeneration and altered pain processing within the primary somatosensory cortex [24]. Reduced PGC-1α in the cortex leads to insufficient control of excitatory neuronal signaling [24]. Research by Wu et al. (2024) showed that wild-type adult mice recovered from nerve-injury-induced mechanical and thermal hypersensitivity, whereas PGC-1α+/− mice failed to resolve these nociceptive behaviors [24]. Both genotypes showed increased calcium activity at day 7 post-injury, but persistent hyperactivation was observed only in PGC-1α+/− haploinsufficient mice [24]. In the cortex, reduced PGC-1α is associated with persistent hyperactivity and pain chronification, while in peripheral pain models, downregulation of PGC-1α directly influences mitochondrial dysfunction [24,68]. These findings suggest a causal role for PGC-1α in pain resolution by maintaining mitochondrial homeostasis and limiting oxidative stress across central as well as peripheral pathways [24,68].

Osteoarthritis pain, characterized by joint damage, involves spinal mitochondrial dysfunction and central sensitization [91]. Earlier work by Sun et al. (2022) indicated that upregulating spinal Sestrin2 (Sesn2) alleviates chronic pain via induction of AMPK/PGC-1α signaling to stimulate mitochondrial generation in spinal neurons [91]. Zhao et al. (2025) showcased that stromal cell-derived factor 1 (SDF-1) relieved osteoarthritis by activating PGC-1α in chondrocytes [92]. The cytoprotective action of SDF-1 on chondrocytes coincides with the elevation of SIRT3 and PGC-1α levels. However, the loss of protection following SIRT3 or mitochondrial inhibition demonstrates that SDF-1 indirectly modulates PGC-1α through upstream SIRT3 signaling [92]. Similarly, with this evidence, Li et al. (2024) showed that prenatal prednisone exposure (PPE) increased long-term osteoarthritis susceptibility in female offspring by suppressing PGC-1α in fetal cartilage [79]. PPE induced hypermethylation of the PGC-1α promoter, impaired mitochondrial biogenesis and glutamine metabolism, and contributed to chondrodysplasia. Notably, pharmacological restoration of PGC-1α expression with ZLN005 reversed these mitochondrial defects and rescued cartilage matrix synthesis in the osteoarthritis model. These findings provide evidence that PGC-1α suppression contributes to PPE-associated cartilage dysfunction and osteoarthritis susceptibility [79].

Post-traumatic osteoarthritis (PTOA) is accompanied by quadriceps wasting, which can worsen joint instability and accelerate cartilage breakdown. A combined human and rabbit study by Liu et al. (2024) demonstrated that joint distraction paired with treadmill exercise reduced inflammatory responses within the joint space and protected cartilage, thereby delaying post-traumatic osteoarthritis development [76]. They also found that lower skeletal muscle PGC-1α levels were associated with more severe osteoarthritis. The quadriceps biopsies from patients with osteoarthritis also showed reduced PGC-1α mRNA and protein expression, supporting an association between muscular PGC-1α deficiency and muscle dysfunction. In the rabbit PTOA model, joint instability downregulated muscle PGC-1α and increased the expression of the atrophy-related markers Atrogin-1 and MuRF1. Exercise and joint distraction restored PGC-1α expression and suppressed these atrophy markers, whereas ZLN005, a PGC-1α activator, produced similar protective effects. These findings support a potential role for reduced skeletal-muscle PGC-1α signaling in PTOA-associated muscle atrophy [76]. Table 1 summarizes PGC-1α dysregulation in musculoskeletal pain.

### 5.2. Peripheral Nerve Injury

Peripheral nerve injury models, including chronic constriction injury (CCI), spinal nerve ligation (SNL), and spared nerve injury (SNI), highlight the ability of PGC-1α to stimulate mitochondrial synthesis and mitigate nerve-injury-induced hypersensitivity [75,91]. However, within the injured peripheral nerves, the biological consequences of PGC-1α dysregulation differ across spinal neurons, microglia, dorsal root ganglion neurons, and immune cells [21,23,41,72,75,78,94,95]. Therefore, the mechanism of PGC-1α’s causal role in chronic pain should be considered a strength of evidence.

In the spinal cord, PGC-1α appears to support mitochondrial biogenesis and redox homeostasis in nociception [91]. Sun et al. (2021) showed that mitochondrial biogenesis within spinal tissue is altered in association with CCI-induced neuropathic hypersensitivity [41]. Under this experimental design, application of the Nrf2 activator RTA-408 upregulated PGC-1α and mitigated mechanical hypersensitivity and thermal nociceptive responses. The PGC-1α activator ZLN005 also produced significant analgesia. This effect, and that of RTA-408, was lost when PGC-1α was pharmacologically inhibited, supporting a causal role of PGC-1α in RTA-408-induced mitochondrial antinociception [41]. These results indicate that RTA-408 acts as an indirect PGC-1α modulator, exerting its antinociceptive effects through Nrf2 activation [41].

In brain disorders, heat shock protein 22 (HSP22) is involved in driving mitochondrial renewal and protecting cells against oxidative damage and neuronal loss [96]. Notably, a marked reduction in HSP22 in spinal neurons has been linked to the progression of neuropathic pain behaviors. Moreover, intrathecal recombinant HSP22 (rhHSP22) attenuated both mechanical hypersensitivity and thermal pain responses in SNI rat models. Accordingly, rhHSP22 lowered ROS levels via the stimulation of spinal AMPK/PGC-1α signaling. Importantly, PGC-1α knockdown with siRNA abolished the analgesic effects of rHSP22. Overall, these findings provide causal evidence that PGC-1α is required for HSP22-mediated protection in neuropathic nociception. Furthermore, inhibiting AMPK with Compound C blocked the rhHSP22-induced improvements in mechanical allodynia, thermal hyperalgesia, and mitochondrial biogenesis. Thus, HSP22 appears to modulate PGC-1α signaling indirectly via AMPK/PGC-1α activation [97].

PGC-1α also influences neuroimmune signaling in the spinal dorsal horn [21]. Chen et al. (2025) reported that spinal PGC-1α activation alleviated CCI-induced neuropathic pain and was associated with interruption of a reciprocal cycle involving mitochondrial malfunction and NLRP3 inflammasome activation [21]. In the spinal dorsal horn, CCI increased pro-inflammatory CD68/IBA1 expression and decreased ARG1/IBA1 microglial expression, while also altering inflammatory cytokine levels. PGC-1α activation restored mitochondrial homeostasis and suppressed NLRP3 inflammasome signaling to restrain microglial neuroinflammation in CCI [21]. Consistently, Zhou and colleagues showed that metformin acted at the injured peripheral sciatic nerve, where it increasedAMPK/PGC-1α/PPAR-γ signaling and reduced CD86, TNF-α, IL-1β, and inducible nitric oxide synthase (iNOS) [94]. The molecular changes were associated with enhanced nerve regeneration and remyelination, as indicated by increasing NF200 and myelin basic protein levels. This evidence suggests that metformin may indirectly influence PGC-1α-associated mitochondrial pathways, thereby contributing to the regulation of spinal neuroinflammation and nerve repair [94].

Additional evidence supports a role for PGC-1α in spinal mitochondrial adaptation and neuroprotection. The selective 5-HT_1F_ receptor agonist lasmiditan alleviated SNI-induced pain through PGC-1α-dependent induction of spinal mitochondrial biogenesis. Importantly, pharmacological inhibition of PGC-1α with SR-18292 reversed the analgesic effect of lasmiditan, suggesting that PGC-1α signaling contributes to lasmiditan-mediated pain relief [78]. These observations are consistent with findings by Simmons and co-workers, who demonstrated that administration of the selective 5-HT_1F_ receptor agonist LY344864 upregulated spinal cord PGC-1α mRNA levels and enhanced functional recovery following spinal cord injury [98]. This bioenergetic recovery suggests that 5-HT_1F_ receptor agonists exert neuroprotective effects by indirectly modulating PGC-1α through 5-HT1F receptor signaling, thereby attenuating nerve-injury-induced pain [78,98].

ChREBP was also reported to promote PGC-1α transcription in spinal microglia, thereby suppressing neuroinflammation and neuronal hyperexcitability in neuropathic pain. In this model, ChREBP knockdown exacerbated mechanical allodynia, whereas ChREBP overexpression alleviated pain. Mechanistic analyses showed that ChREBP enhanced PGC-1α transcription by binding to its promoter. Moreover, PGC-1α overexpression rescued the inflammatory and neuronal changes induced by ChREBP knockdown. These findings support a mechanistic role for PGC-1α in the protective effects of ChREBP against neuropathic pain [95].

In the CNS, mitochondrial outer membrane translocator protein (TSPO) has shown promise in alleviating neuropathic pain through the regulation of mitochondrial activity. Ro5-4864, a TSPO agonist, relieved mechanical hypersensitivity in association with increased PGC-1α signaling, improved mitochondrial activity, and reinforced antioxidant protection [72]. Consistent with these findings, Li et al. (2025) established that increasing TSPO levels mitigated SNI-driven neuropathic pain through restored mitochondrial function and reduced pyroptosis in dorsal horn neurons [99]. Pharmacological upregulation of TSPO also increased PGC-1α and conferred antinociceptive actions. However, blocking PGC-1α pharmacologically abolished the beneficial effects of TSPO on mitochondrial balance and pyroptosis, providing evidence that PGC-1α contributes to TSPO-mediated antinociception [99]. These data indicate that TSPO and Ro5-4864 indirectly modulate PGC-1α through downstream TSPO signaling and mitochondrial pathway regulation [72,99].

Epigenetic mechanisms also contribute to neuropathic pain by repressing antinociceptive genes [23]. PGC-1α can be suppressed transcriptionally during neuropathic pain through DNA methylation, histone modification, and chromatin remodeling [100]. RNA-seq analysis identified Sp1 as related to CCI-provoked neuropathic pain and predicted its binding to the PGC-1α promoter. Spinal Sp1 upregulation repressed PGC-1α expression by recruiting histone deacetylase 2 (HDAC2) to the PGC-1α promoter. Silencing Sp1 reduced inflammation, mitigated mitochondrial dysfunction in the spinal cord, and improved pain sensitivity. Likewise, overexpression of PGC-1α ameliorated CCI-provoked neuropathic pain and mitochondrial dysfunction [23]. This points to epigenetic regulators such as Sp1 or HDAC2 as potential targets to prevent PGC-1α silencing in chronic neuropathic pain [23].

Additional evidence has suggested that stimulating G protein-coupled receptor 39 (GPR39) recruits PGC-1α to drive mitochondrial biogenesis and mitigate mechanical allodynia. The effects of GPR39 activation were abolished by PGC-1α siRNA, providing evidence for a PGC-1α-dependent analgesic mechanism in the SNI model [75]. Dietary ketosis increased spinal dorsal horn PGC-1α levels and was associated with reduced CCI-triggered mechanical and thermal hypersensitivity. [101]. Melatonin inhibited SNL-induced neuropathic pain in association with increased PGC-1α expression and improved mitochondrial membrane potential in dorsal root ganglia [102]. Another study found that bioactive molecules in turmeric alleviate mechanical hypersensitivity in association with reduced glial activation and restored mitochondrial function across both the spinal cord and amygdala [103]. These studies show that GPR39 activation, dietary ketosis, melatonin, and turmeric bioactive compounds indirectly modulate PGC-1α through SIRT1/PGC-1α upstream signaling, metabolic regulation, and mitochondrial pathways [75,101,102,103].

In essence, across CCI, SNI, and SNL models, reduced PGC-1α is consistently associated with mitochondrial malfunction, oxidative damage, and persistent neuropathic pain [97,101,102]. Sp1/HDAC2 provide epigenetic repression of PGC-1α, thereby promoting a pro-nociceptive state [23]. Activation of PGC-1α by Nrf2, TSPO, AMPK, 5-HT1F, GPR39, HSP22, ChREBP, metformin, melatonin, and turmeric restores mitochondrial biogenesis in pain models [72,78,91,95,97,102]. However, these interventions produce biological effects through various molecular targets and function as indirect PGC-1α modulators rather than direct PGC-1α agonists [104,105,106]. Therefore, increased PGC-1α expression alone cannot establish that PGC-1α mediates the antinociceptive effects. The stronger evidence supporting PGC-1α as a causal regulator of neuropathic pain comes from studies in which its manipulation changes mitochondrial function and pain behavior using PGC-1α inhibition, knockdown, overexpression, or rescue approaches [23,97,99,107]. A summary of PGC-1α dysregulation in peripheral nerve injury is provided in Table 2.

### 5.3. Chemotherapy-Induced Neuropathic Pain

In a rat model, paclitaxel-induced neuropathic pain (PINP) was found to impair mitochondrial biogenesis in the nociceptive system, with reduced levels of PGC-1α in the CNS. Moreover, formoterol enhanced PGC-1α expression and blocked mechanical allodynia. These findings suggest that the β2-adrenergic system may relieve chemotherapy-induced neuropathic pain in association with PGC-1α-related mitochondrial renewal [107]. Recently, Mo and colleagues (2026) documented that pituitary adenylate cyclase-activating polypeptide (PACAP) relieved PINP through a PGC-1α-dependent mechanism. PACAP upregulated PGC-1α, reduced oxidative damage, and attenuated mechanical allodynia and hyperalgesia. Pharmacological inhibition of PGC-1α reversed these effects, supporting a causal role for PGC-1α in PACAP-mediated pain relief by improving mitochondrial homeostasis [80].

### 5.4. Cancer-Induced Bone Pain

In a rodent model of cancer-provoked bone pain, Ge et al. (2022) reported that polarization of spinal microglia was associated with suppressed PGC-1α and reduced glutathione peroxidase 4 (GPx4) [85]. Intraperitoneal naringenin administration relieved bone cancer-induced bone pain-related mechanical allodynia and promoted spinal GPx4 expression via the AMPK/PGC-1α system [85]. Chen et al. (2025) supported this mechanism by showing that spinal PGC-1α activation mitigated neuropathic pain by reprogramming microglia [21]. Notably, a reduction in PGC-1α expression was observed in spinal GABAergic interneurons. Treatment with the intrathecally administered PGC-1α agonist ZLN005 preserved GABAergic interneuron populations, alleviated mechanical allodynia, and restored mitochondrial biogenesis. These biological effects were reversed by the PGC-1α antagonist SR-18292, indicating that PGC-1α-dependent mitochondrial protection in inhibitory interneurons plays a role in limiting cancer-induced bone pain [25].

### 5.5. Diabetes-Induced Neuropathic Pain

Diabetic nerve injury is marked by oxidative stress and impairment in PGC-1α-dependent mitochondrial biogenesis. In painful diabetic neuropathy, suppression of this pathway is associated with mitochondrial abnormality and peripheral nerve damage, particularly in Schwann cells, sciatic nerve tissue, and dorsal root ganglion neurons [81,82,83,84]. In diabetic peripheral neuropathy, quercetin was associated with increased PGC-1α activity in sciatic nerves, dorsal root ganglion neurons, and Schwann cells [84]. Similarly, the mitochondria-derived peptide MOTS-c increased PGC-1α expression in the spinal dorsal horn and was associated with improved mitochondrial health and reduced hyperalgesia in streptozotocin-induced painful diabetic neuropathy [82]. Tang Bi formula was associated with modulation of PGC-1α signaling, improved mitochondrial balance, and attenuation of axonal degeneration in sciatic nerves and Schwann cells [83]. These studies support PGC-1α as a potential metabolic target through which neuroprotective agents could preserve mitochondrial integrity, improve redox balance, and attenuate nerve injury in diabetic neuropathy [81,82,83,84]. Table 3 summarizes evidence of PGC-1α dysregulation in chemotherapy-induced neuropathic pain, cancer-induced bone pain, and diabetes-induced neuropathic pain.

### 5.6. Fibromyalgia

Mitochondrial dysfunction in skeletal muscle and hippocampus is coupled to suppression of PGC-1α signaling in fibromyalgia models. This suppression promotes redox imbalance and mitochondrial abnormalities, which contribute to nociplastic pain and cognitive impairment [77,86,87]. Belviranlı and colleagues reported that exercise training and coenzyme Q10 improve fibromyalgia symptoms. These benefits were associated with the indirect stimulation of hippocampal PGC-1α, which improved redox homeostasis and mitochondrial renewal in skeletal muscle fibers [86,87]. Similarly, melatonin restored PGC-1α signaling, mitigated neuronal sensitization, and suppressed neuroinflammation [77,108]. Such evidence highlights that PGC-1α serves as a neuroprotective target mediating the beneficial effects of both pharmacological and non-pharmacological measures, including exercise, coenzyme Q10, and melatonin, in fibromyalgia [77,86,87,108]. A summary of PGC-1α dysregulation in fibromyalgia is provided in Table 4.

### 5.7. Chronic Headache Disorders

Chronic headache disorders, particularly migraines, are associated with mitochondrial malfunction and alteration in PGC-1α-related signaling in trigeminal pathways [74,88]. Prior findings using nitroglycerin-triggered migraine models showed that lower PGC-1α levels correlated with central sensitization and inflammatory pathways in chronic migraine progression [74]. Guan and collaborators demonstrated that mitochondrial dysregulation in the trigeminal ganglion was associated with nociception in nitroglycerin-triggered migraine and impaired PGC-1α expression and mitochondrial health [88]. Similarly, administration of the mitochondrial-targeted peptide SS-31 alleviated pain responses and revived mitochondrial activity, accompanied by increased PGC-1α expression, in an inflammatory soup model [89]. Table 5 summarizes PGC-1α dysregulation in chronic headache disorders.

### 5.8. Opioid-Induced Hyperalgesia

Chronic opioid exposure is associated with opioid-induced hyperalgesia and tolerance through downregulation of PGC-1α-dependent mitochondrial signaling [26,90]. Jie et al. (2025) showed that morphine tolerance correlated with enhanced CCL2 and inhibition of Nrf2/PGC-1α signaling [90]. These changes promote oxidative stress and neuronal apoptosis, thereby reducing analgesic effects. In this study, pharmacological manipulation with the CCL2 inhibitor Bindarit or the PGC-1α transcriptional activator ZLN005 abolished spinal apoptosis and reversed tolerance from morphine administration [90]. Feng et al. (2025) reported a similar pattern in remifentanil-induced hyperalgesia, where activation of spinal p38 MAPK suppressed PGC-1α, promoted neuronal ROS release, thereby contributing to pain hypersensitivity [109]. In line with these results, Kashiwagi et al. (2021) showed that recombinant PGC-1α reduced mitochondrial superoxide and attenuated spinal morphine tolerance [26]. Notably, NeuroAid improved morphine-induced cognitive deficits, potentially by upregulating PGC-1α [110]. The basolateral amygdala SIRT1/PGC-1α pathway and mitochondrial dysregulation were also implicated in morphine withdrawal symptoms [111]. Table 6 summarizes PGC-1α dysregulation in opioid-induced hyperalgesia.

## 6. Opportunities in Targeting PGC-1α for Chronic Pain

PGC-1α represents a potential neurotherapeutic target in chronic pain because of its involvement in regulating mitochondrial health, antioxidant defense, and neuroinflammation [94,96]. In both chronic pain models and neurodegenerative or neurological conditions, reduction in PGC-1α expression plays a key role in neuropathological alterations [64,96]. However, most available evidence has been derived from preclinical samples of the brain, spinal cord, dorsal root ganglia, hippocampus, or injured peripheral nerves, which are not readily accessible for routine assessment in patients [26,80,84,90,107,112]. Skeletal muscle may represent a more accessible tissue for evaluating PGC-1α expression in pain conditions associated with muscle dysfunction such as osteoarthritis and fibromyalgia [76,108,113]. However, whether skeletal muscle PGC-1α activity reflects PGC-1α signaling in central pain pathways remains uncertain. Validating this cross-tissue relationship would help establish whether skeletal muscle PGC-1α can serve as a peripheral biomarker of central mitochondrial dysregulation. This possibility is supported by evidence associating lower skeletal muscle mitochondrial function with cognitive decline and increased dementia risk [114].

Several endogenous and pharmacological molecules increase PGC-1α expression or activity. AMPK promotes PGC-1α signaling in response to cellular energy stress, whereas SIRT1 activates PGC-1α through deacetylation to enhance mitochondrial biogenesis and antioxidant defenses [64,70,71]. PPARγ interacts with PGC-1α to regulate metabolic, mitochondrial, and anti-inflammatory processes [20]. These mechanisms are relevant to chronic pain through dysregulation of the AMPK/SIRT1/PGC-1α and PPAR/PGC-1α signaling pathways, contributing to pain-induced cellular dysfunction [97,115].

Bioactive molecules, including resveratrol, berberine, quercetin, evodiamine, and salvianolic acid A, have been reported to exert neuroprotective effects, in part through the modulation of PGC-1α and improving mitochondrial resilience [84,116,117,118,119]. Resveratrol and quercetin may enhance PGC-1α activity through AMPK/SIRT1 mechanisms, while other phytochemicals may regulate PGC-1α expression through antioxidant, anti-inflammatory, and mitochondrial protective pathways [84,116]. Magnol and polyphenols have also been investigated for their potential to modulate PGC-1α-related pathways [39,120]. Overall, these compounds have been associated with mitochondrial protection and reduction in oxidative stress, supporting their potential relevance for PGC-1α-mediated modulation of pain pathways [103,117,119,120]. In addition, compounds such as mitoquinone, pyrroloquinoline quinone, alpha-lipoic acid, acetyl-L-carnitine, and nicotinamide riboside are promising mitochondrial protectants and antioxidants for further study of PGC-1α in chronic nociception [92,121,122,123]. Together, these compounds promote mitochondrial preservation, reduce oxidative stress, and attenuate neuroinflammation, thereby supporting the rationale for targeting PGC-1α in chronic pain [84,116,117,118,119].

Small-molecule PGC-1α activators and stabilizers such as ZLN005 and other high-throughput screening hits may offer an additional strategy for chronic pain by enhancing PGC-1α expression and mitochondrial biogenesis [27,124], whereas SR-18292 has been reported to inhibit the biological effects of PGC-1α [69]. Neuroprotective agents such as N-acetylcysteine and idebenone may also act as antioxidants, mitochondrial protectants, or neuroinflammation modulators [125,126]. These compounds warrant further investigation for their potential roles in modulating PGC-1α in pain mechanisms.

An additional translational possibility is that PGC-1α-targeted approaches could provide symptomatic relief from opioid-induced tolerance. This is supported by the finding that spinal PGC-1α was downregulated during morphine tolerance, whereas restoration of PGC-1α attenuated mechanical and thermal hypersensitivity in morphine-tolerant rodents [26]. Recent studies also implicate PGC-1α/SIRT3 and Nrf2/PGC-1α impairment in remifentanil-induced hyperalgesia and morphine tolerance, respectively, both of which were reversed by ZLN005 intervention [90,109]. Specifically, dysregulated mitochondrial renewal increases oxidative stress and promotes apoptosis by impairing the Nrf2 pathway [90,109], whereas recombinant PGC-1α reduced morphine-associated mitochondrial superoxide [26]. These findings suggest that PGC-1α activation may counteract morphine-induced neuroadaptations by supporting mitochondrial renewal, reducing mitochondrial oxidative stress, and limiting apoptosis. Accordingly, PGC-1α activation could serve as a candidate strategy for providing symptomatic relief in opioid-induced tolerance and hyperalgesia [90,109]. However, the findings do not establish that PGC-1α-targeted interventions reduce opioid dose requirements. Therefore, future studies should test whether PGC-1α activators can reduce opioid dose requirements.

Chronic pain management sometimes requires combination therapy to improve efficacy and limit opioid adverse effects [127]. Future studies should investigate whether PGC-1α activators can enhance the therapeutic efficacy of opioids. Direct comparisons of PGC-1α activators with opioid analgesics are also required to evaluate their potential as standalone non-opioid options. In addition, PGC-1α-targeted approaches show promising analgesic effects in preclinical studies [116]. However, direct head-to-head comparisons with currently used analgesics such as NSAIDs, opioids, anticonvulsants, or antidepressants are limited. Future research should evaluate their efficacy relative to standard analgesics and determine their potential as adjunctive or opioid-sparing therapies.

## 7. Challenges and Limitations in Targeting PGC-1α for Chronic Pain

Despite its compelling role in mitochondrial function and pain modulation, targeting PGC-1α for chronic pain presents several biological, pharmacological, and translational limitations. A major translational challenge in targeting PGC-1α is achieving tissue- and cell-type-specific modulation while avoiding systemic effects, given the broad expression of PGC-1α and its inducible regulation of mitochondrial and inflammatory pathways [64,66,128,129,130]. The biological effects of PGC-1α activation are likely to depend on its level, duration, and tissue [131,132].

Preclinical findings indicate that excessive or sustained PGC-1α activation has potential adverse effects and safety concerns. For instance, PGC-1α overexpression improved cardiac function in young wild-type rodents but accelerated aging in older mice [133]. In transgenic mice, elevated cardiac PGC-1α expression induced abnormal mitochondrial proliferation and dilated cardiomyopathy [134]. Consistent with these observations, PGC-1α overexpression increased left-ventricular dilation and mortality following transverse aortic constriction, suggesting that persistent stimulation of PGC-1α may compromise cardiac adaptation to stress [135].

While physiological PGC-1α activity contributes to metabolic homeostasis, excessive expression has been associated with insulin resistance [136,137]. In a PGC-1α transgenic mouse model, elevated PGC-1α increased the risk of diet-induced insulin resistance. This effect potentially stemmed from impaired insulin-induced glucose uptake and increased intracellular lipid accumulation [138]. In addition, prolonged PGC-1α overexpression in the nigrostriatal pathway produced a dose-dependent loss of dopaminergic markers and depletion of striatal dopamine. Also, the higher expression levels produced dopaminergic neurodegeneration [139]. Before clinical translation for chronic pain, future studies should evaluate the therapeutic window, treatment duration, CNS bioavailability, and long-term cardiovascular, metabolic, and neurological safety of PGC-1α-targeted therapies.

Sex-related biological variation may further complicate targeting PGC-1α because males and females may respond differently [140]. For example, exercise-induced PGC-1α expression appears to be sex-dependent. Moreover, cardiac studies suggest that PGC-1α may contribute to sex-specific variation in cardiac function and cardiovascular risk [140]. Recent studies also show that PGC-1α-dependent thermogenesis in brown adipose tissue is influenced by sex [141]. This is concerning because chronic pain, including fibromyalgia and migraine, is more common in females [142,143]. These findings could have implications for chronic pain because peripheral metabolic and inflammatory tissues communicate with the CNS through circulating cytokines, adipokines, and neural pathways, thereby influencing neuroinflammation and pain processing [144,145].

Specifically, sex representation varied across fibromyalgia models, with several reserpine-induced studies using female Wistar rats [77,86,112], while another study used male Sprague-Dawley rats [108]. In contrast, studies of nitroglycerin-induced migraine used male mice [74,88], whereas the inflammatory-soup headache study was predominantly male, with limited female inclusion [89]. Although these studies include both male and female animals, differences in pain model and experimental design limit direct comparisons between sexes. Therefore, a lack of head-to-head comparisons between males and females within the same experimental design remains a key scientific gap. [74,77,86,88,89,108,112]. Given that PGC-1α is sex-dependent, this gap warrants direct attention.

Natural molecules such as curcumin and berberine have shown neuroprotective effects in preclinical studies by targeting PGC-1α [28,146,147]. However, these molecules influence other signaling pathways, including AMPK and Nrf2 [64]. Therefore, their effects may not be attributable solely to direct PGC-1α activation [148]. Clinically used compounds such as melatonin and metformin appear promising for PGC-1α-centered therapy [77,149]. However, more studies are required to define the optimal dosing and translational potential of metformin–melatonin combinations in pain models [149]. In addition, the small-molecule PGC-1α agonist ZLN005 has shown activity in mouse models, whereas SR-18292 has been documented to inhibit PGC-1α activity in animal studies [69]. However, variations in chronic pain models and the species gap between rodents and humans remain major barriers to clinical translation [150]. Finally, PGC-1α serves as a regulator of gene expression, coactivating transcription factors rather than a classic receptor, making direct pharmacological targeting particularly challenging [129]. Future research should consider modulating PGC-1α through post-translational protein modifications alongside interactions with target proteins to influence its stability and activity [20,27,129]. Furthermore, structural biology could potentially identify functional domains of PGC-1α for future structure-based drug design. These limitations highlight that substantial future research is required to translate PGC-1α modulation into safe, effective, and sex-specific clinical therapies for chronic pain.

## 8. Conclusions and Future Directions

PGC-1α appears to be a promising disease-modifying neuroprotective target in chronic pain by modulating mitochondrial dysfunction, oxidative stress, and neuroinflammation. However, its effects vary across tissues and pain conditions. Natural compounds, including resveratrol, berberine, quercetin, evodiamine, and salvianolic acid A; some clinically used drugs; and synthetic small-molecule modulators show potential as PGC-1α-targeting analgesic agents. However, most findings remain preclinical, with challenges related to sex specificity, tissue specificity, and limited translation to humans. Future scientific work should define the role of PGC-1α in sex-specific pain models and optimize dosing to develop more selective, effective, and tolerable analgesics.

The development of lipid nanoparticles or blood–brain-barrier-penetrating formulations will be important for directing PGC-1α activators to the CNS. This is because targeted delivery systems could help reduce systemic side effects by limiting off-target exposure and improving CNS delivery. Future preclinical work should determine which types of chronic pain benefit most from PGC-1α modulation. Combining PGC-1α modulators with low-dose opioids may reduce tolerance, as PGC-1α stimulation has been demonstrated to mitigate both morphine tolerance and chronic pain. In addition, future studies should investigate whether PGC-1α upregulation can also mitigate other major opioid-related adverse effects, such as respiratory depression, gastrointestinal dysmotility, and physical dependence or withdrawal syndromes.

## Figures and Tables

**Table 1:** PGC-1α dysregulation in musculoskeletal pain.

Pain Model/References	PGC-1α Expression	Tissue/Cell Affected	Effects Associated with PGC-1α Dysregulation	Biological Effects of PGC-1α Restoration
Aging-associated pain after nerve injury [24]	Age-related decline in cortical PGC-1α	Somatosensory cortex GABAergic interneurons	Persistent excitatory overactivity	Restoration of cortical dynamics and reversal of chronification
Osteoarthritis [91,92,93]	Reduced AMPK/PGC-1α- or Sirt3/PGC-1α-related signaling	Spinal dorsal horn neurons and osteoarthritic joint tissue	Mitochondrial dysfunction, oxidative stress, spinal gliosis, cartilage degeneration, and pain-related behaviors	Sesn2, dimethyl fumarate, and SDF-1 were associated with modulation of PGC-1α-related signaling and reduced pain
Post-traumatic osteoarthritis and muscle wasting [76]	PGC-1α downregulation in skeletal muscle	Quadriceps skeletal muscle	Muscle atrophy, inflammation, and cartilage loss	Joint distraction and treadmill exercise restored PGC-1α and suppressed muscle wasting
Myofascial pain syndrome [68]	Downregulation of the AMPK/PGC-1α/SIRT3 axis	Skeletal muscle	Depressed mitochondrial function and myofascial pain	Restoration of the AMPK/PGC-1α/SIRT3 axis was associated with improved mitochondrial health and reduced myofascial pain

**Table 2:** PGC-1α dysregulation in peripheral nerve injury.

Pain Model/Reference	PGC-1α Expression	Tissue/Cell Affected	Effects Associated with PGC-1α Dysregulation	Biological Effect of PGC-1α Restoration
CCI-triggered neuropathic pain [41]	Spinal PGC-1α, NRF1, TFAM, and mtDNA were reduced.	L4-L6 spinal cord dorsal horn.	Decrease in mitochondrial biogenesis and hyperalgesia.	RTA-408 and ZLN005 increased PGC-1α and alleviated pain.
[21]	Spinal PGC-1α activity was reduced.	Dorsal horn microglia.	Mitochondrial dysfunction and NLRP3 activation.	PGC-1α activation restored mitochondrial function, shifted microglia toward an anti-inflammatory phenotype, and reduced pain.
[23]	PGC-1α transcription was repressed by Sp1/HDAC2.	Spinal cord microglia and neurons.	Mitochondrial disruption, oxidative damage, and neuronal dysfunction.	PGC-1α overexpression reversed mitochondrial dysfunction and alleviated pain.
[101]	PGC-1α was reduced and restored by a ketogenic diet.	Spinal dorsal horn microglia.	Neuroinflammation, ROS accumulation, and mitochondrial dysregulation.	Ketogenic diet increased PGC-1α, improved mitochondrial function, and relieved pain.
Peripheral nerve injury [94]	AMPK/PGC-1α/PPAR-γ signaling was reduced in macrophages.	Bone-marrow-derived macrophages at the injury site.	Increased iNOS/TNF-α/IL-1β, impaired axon regeneration and remyelination.	Metformin activated PGC-1α and improved regeneration and recovery.
SNI-induced neuropathic pain [97]	Spinal AMPK/PGC-1α signaling was impaired.	Spinal dorsal horn.	Reduced NRF1/TFAM-dependent mitochondrial biogenesis and increased ROS/oxidative stress.	rhHSP22 activated PGC-1α, restored mitochondrial biogenesis, and alleviated hypersensitivity.
[78]	Spinal PGC-1α signaling was impaired.	Lumbar dorsal horn neurons.	Reduced mitochondrial biogenesis and persistent mechanical allodynia.	Lasmiditan activated PGC-1α, restored mitochondrial biogenesis, and reduced allodynia.
[99]	PGC-1α signaling was reduced.	Spinal dorsal horn astrocytes.	Mitochondrial dysfunction, disrupted dynamics, pyroptosis, and inflammation.	TSPO activation restored PGC-1α, improved mitochondrial health, and reduced pain.
[95]	ChREBP increased PGC-1α transcription.	Spinal dorsal horn microglia.	Reduced PGC-1α lowered fatty acid oxidation and promoted pro-inflammatory effects.	PGC-1α restored microglial fatty acid oxidation, reduced cytokines, and pain.
[75]	SIRT1/PGC-1α signaling and GPR39 expression were reduced.	Spinal dorsal horn neurons and microglia.	Mitochondrial dysfunction, neuroinflammation, and persistent allodynia.	TC-G 1008 activated PGC-1α, improved mitochondrial function, and reduced pain.
SNL-induced neuropathic pain [102]	SIRT1 and PGC-1α were reduced in DRG neurons.	L5-L6 dorsal root ganglion neurons.	Mitochondrial dysregulation and oxidative stress.	Melatonin restored PGC-1α expression, improved mitochondrial biogenesis, and reduced pain.
[72]	Nuclear SIRT1/PGC-1α signaling was reduced.	Spinal dorsal horn.	Abnormal mitochondrial biogenesis, high oxidative stress, and mechanical allodynia.	Ro5-4864 restored PGC-1α, improved mitochondrial biogenesis, and reduced allodynia.

**Table 3:** PGC-1α dysregulation in chemotherapy-induced neuropathic pain, cancer-induced bone pain, and diabetes-induced neuropathic pain.

Pain Model/Reference	PGC-1α Expression	Tissue/Cell	Effects Associated with PGC-1α Dysregulation	Biological Effect of PGC-1α Restoration
Paclitaxel-induced neuropathic pain [107]	PGC-1α-mediated mitochondrial biogenesis was induced in the spinal cord.	Spinal cord	Impaired mitochondrial biogenesis contributed to mechanical allodynia.	β2-adrenoreceptor activation restored PGC-1α and alleviated mechanical allodynia.
[80]	PGC-1α was expressed in dorsal root ganglion.	Dorsal root ganglion	Oxidative stress and mitochondrial damage promoted peripheral neuropathy.	PACAP activated PGC-1α, enhanced mitochondrial health, reduced oxidative damage, and alleviated neuropathy.
Cancer-induced bone pain [85]	PGC-1α signaling was suppressed.	Spinal microglia	M1 polarization, NF-κB activation, and reduced GPx4.	Naringenin activated PGC-1α, promoted M2 polarization, and alleviated pain.
[25]	PGC-1α was downregulated in the spinal cord.	Spinal GABAergic interneurons	Interneuron apoptosis, mitochondrial dysfunction, increased ROS, and allodynia.	ZLN005 restored PGC-1α, preserved interneurons, and reduced allodynia.
Diabetic peripheral neuropathy [84]	PGC-1α was suppressed.	Sciatic nerves, dorsal root ganglia neurons, and Schwann cells	Mitochondrial dysfunction and oxidative stress.	Quercetin activated PGC-1α and improved neuropathy.
Streptozotocin-induced painful diabetic neuropathy [82]	PGC-1α expression was induced by a mitochondria-derived peptide (MOTS-c).	Spinal dorsal horn	Mitochondrial dysfunction with hyperalgesia and allodynia.	MOTS-c activated PGC-1α, improved mitochondrial health, and alleviated pain.
[83]	Suppression of PGC-1α signaling.	Sciatic nerves and Schwann cells	Mitochondrial imbalance, axonal degeneration, and oxidative stress.	Tang Bi formula activated PGC-1α and improved nerve injury.

**Table 4:** PGC-1α dysregulation in fibromyalgia.

Pain Model/Reference	PGC-1α Expression	Tissue/Cell Affected	Effects Associated with PGC-1α Dysregulation	Biological Effect of PGC-1α Restoration
Reserpine-induced fibromyalgia [86]	Alteration in hippocampal PGC-1α.	Hippocampus	Cognitive decline, reduced locomotion, increased depression-like behavior, and mechanical allodynia.	Exercise training activated hippocampal PGC-1α and improved fibromyalgia symptoms.
[87]	Reduction in PGC-1α expression.	Gastrocnemius and soleus muscle	Redox imbalance, reduced mitochondrial biogenesis, and fibromyalgia-like pain.	CoQ10 restored redox homeostasis and increased PGC-1α.
[87]	PGC-1α signaling was altered.	Hippocampus	Fibromyalgia-like symptoms, cognitive deficits, and impaired mitochondrial and redox homeostasis.	CoQ10 enhanced hippocampal PGC-1α signaling and improved symptoms and cognition.
[108]	PGC-1α and CoQ10 were reduced.	Gastrocnemius muscle	Mitochondrial dysfunction and oxidative stress.	Melatonin preserved mitochondrial health and prevented oxidative stress via PGC-1α signaling.
[77]	PGC-1α signaling was suppressed.	Spinal cord	Central sensitization and nociplastic pain, with MAPK/NF-κB activation.	Melatonin restored PGC-1α signaling and reduced pain sensitization.

**Table 5:** PGC-1α dysregulation in chronic headache disorders.

Pain Model/Reference	PGC-1α Expression	Tissue/Cell Affected	Effects Associated with PGC-1α Dysregulation	Biological Effect of PGC-1α Restoration
Nitroglycerin-induced chronic migraine [74]	Downregulation of PGC-1α.	Trigeminal nucleus.	Inflammation and central sensitization.	SIRT1 overexpression or SRT1720 alleviated inflammation and pain sensitization.
[88]	Decreased PGC-1α expression.	Trigeminal ganglion neurons.	Mitochondrial dysfunction, periorbital allodynia, and mechanical hyperalgesia.	*Schisandra chinensis* and mitoquinone improved mitochondrial function and alleviated migraine pain.
Inflammatory-soup-induced headache model [89]	Reduction in PGC-1α expression.	Trigeminal nucleus caudalis.	Nociceptive responses and mitochondrial dysfunction.	SS-31 abolished nociception and restored mitochondrial function.

**Table 6:** PGC-1α dysregulation in opioid-induced hyperalgesia.

Pain Model/References	PGC-1α Expression	Tissue/Cell Affected	Effects Associated with PGC-1α Dysregulation	Biological Effect of PGC-1α Restoration
Morphine tolerance [90]	Reduced PGC-1α-expression.	Spinal cord.	Nrf2 inhibition and mitochondrial dysfunction.	PGC-1α activator ZLN005 protected against spinal cord apoptosis and morphine tolerance.
[26]	PGC-1α suppressed spinal morphine tolerance.	Spinal cord.	Increased mitochondrial superoxide and tolerance.	Recombinant PGC-1α reduced mitochondrial superoxide and abolished morphine tolerance.
Remifentanil-induced hyperalgesia [109]	p38 MAPK/PGC-1α/SIRT3 expression was suppressed.	Spinal cord.	ROS release and hyperalgesia.	Modulating p38 MAPK/PGC-1α signaling reduced hyperalgesia.

## Data Availability

No data were generated or analyzed in this manuscript. Data sharing is not applicable to this article.

## Abbreviations

The following abbreviations are used in this manuscript:

AMPK	AMP-activated protein kinase
CAT	catalase
CCI	chronic constriction injury
CNS	central nervous system
ERRs	estrogen-related receptors
GPX1	glutathione peroxidase 1
GPR39	G protein-coupled receptor 39
GPx4	glutathione peroxidase 4
HDAC2	histone deacetylase 2
HSP22	heat shock protein 22
mtDNA	mitochondrial DNA
mtROS	mitochondrial reactive oxygen species
NF-κB	nuclear factor kappa-light-chain-enhancer of activated B cells
NSAIDs	non-steroidal anti-inflammatory drugs
NRF	nuclear respiratory factor
PACAP	pituitary adenylate cyclase-activating polypeptide
PGC-1α	PPAR gamma coactivator-1α
PINP	paclitaxel-induced neuropathic pain
PPAR	peroxisome proliferator-activated receptor
PPE	prenatal prednisone exposure
PTOA	post-traumatic osteoarthritis
rhHSP22	recombinant heat shock protein 22
ROS	reactive oxygen species
SDF-1	stromal cell-derived factor 1
SNI	spared nerve injury
SNL	spinal nerve ligation
SOD2	superoxide dismutase 2
SS-31	Szeto–Schiller peptide 31
TSPO	translocator protein
